# Analysis of Motorcyclist Riding Behaviour on Speed Table

**DOI:** 10.1155/2014/236396

**Published:** 2014-06-01

**Authors:** Choon Wah Yuen, Mohamed Rehan Karim, Ahmad Saifizul

**Affiliations:** Centre for Transportation Research, University of Malaya, 50603 Kuala Lumpur, Malaysia

## Abstract

This paper focuses on the study of the change of various types of riding behaviour, such as speed, brake force, and throttle force applied, when they ride across the speed table. An instrumented motorcycle equipped with various types of sensor, on-board camera, and data logger was used in acquiring the traffic data in the research. Riders were instructed to ride across two speed tables and the riding data were then analyzed to study the behaviour change from different riders. The results from statistical analysis showed that the riding characteristics such as speed, brake force, and throttle force applied are influenced by distance from hump, riding experience, and travel mileage of riders. Riders tend to apply higher brake intensity at distance point 50 m before the speed table and release the braking at point −10 m after the hump. In short, speed table has different rates of influence towards riding behaviour on different factors, such as distance from hump and different riders' attributes.

## 1. Introduction

Since Malaysia economy is booming, the number of road users increased sharply in the last decade. Each year, the numbers of traffic accidents that were recorded in Malaysia are standing very high. It is well recognized that road safety is a public health problem as road traffic accidents are among the eight leading causes of death worldwide, according to Loo et al. [1]. In Malaysia, motorcycles constitute more than half the total vehicle population and contribute more than 47% of the casualties (deaths and serious and slight injuries) in traffic crashes, which recorded 48%, 47.4%, and 47.7% for the year 2006, 2007, and 2008, respectively. Due to high casualty's rate from motorcyclist, the need to create policy in improving safety of this transport mode becomes an important issue nowadays. Studies on accident trends in the country have shown that the rapid increase in level of motorization and growth in motorcycle population has also contributed towards the increase in accidents involving motorcyclists [2, 3]. According to Radin Umar et al. [4], a preliminary investigation on motorcycle fatalities showed that riding a motorcycle is 17 times more dangerous than driving a passenger car. It can be inferred that in the event of a traffic accident involving a motorcyclist and other motorized vehicle, the motorcyclist will be facing higher risk of injury or fatality because the degree of exposure to injury is higher for the motorcyclist as compared to vehicle occupant in the event of a crash. Thus, safety of this form of transportation is an important issue.

Motorcycle accident study has always been an active research topic in the recent years. These studies mostly focus on the causes and factors of traffic accidents. Research on the crash risk of motorcyclists has investigated a variety of issues, such as rider attributes, motorcycle characteristics, roadway, environmental and traffic factors, and overexposure of motorcycles at intersections [5]. Chang and Yeh [6] investigation shows that young and male motorcycle riders seem to have more accidents due to risky and violation behaviour. Besides, female riders also found involved in an accident due to other latent factors such as lack of experience and skills. Broughton et al. [7] found that riders who ride at unsafe high speed will have higher crash probability. Furthermore, a study also showed that greater proportions of both young and elderly drivers lead to higher death rates [8]. Accidents statistics also proved that young drivers have significantly higher accident violation rates than older drivers. This is further supported in Wong et al. [9] study, where they had identified young motorcyclist as high-risk population causing motorcycle accidents. Steg and van Brussel [10] had found that speeding violations were the most common aberrant behaviour among the motorcyclists. Young motorcycle riders may not be fully aware of the errors, lapses, and violations they make and may not provide accurate assessments of their aberrant behaviour. Charles et al.'s [11] study shows that young drivers have significantly higher accident violation rates than older drivers. In short, many previous studies have proved that young riders seem to expose more in the motorcycle crash [6, 8–12].

One of the reasons which found significant on accident occurrence is overspeeding. Overspeeding may increase the potential of accident occurrence. This is because the travel roadway was not designed to accommodate speeds that exceeded the roadway design speed. Speed limits and speed enforcement have been major policy initiatives of many road safety strategies [13]. It is observed that, generally, motorists do not adhere to speed limits but instead choose speeds they perceives acceptably safe [14]. Thus, some devices need to be installed in order to calm the vehicular traffic and make the road environment safer for everyone. One of the common methods to calm vehicular traffic is to have a speed calming measure. A speed calming measure involves modifying the physical features of traveled roadway so that vehicles are forced to slow down when they come across the particular road path. One of the most common speed calming measures that is practiced in Malaysia is the road hump. The main purpose of the road hump is to ensure that vehicle speed is reduced to an acceptable level at a certain location along a road. The overall operating speed of the vehicle on that road can be low enough for the vehicle to stop safely, thus avoiding a crash. A road hump may be used individually or in combination with other facilities such as a pedestrian crossing. Different road humps with a variety of cross-sectional geometry not only help in reducing vehicular speed but also have impacts on the comfort of the driver and the vehicle occupants. The design of this study focuses on the effectiveness of speed table in reducing speed and the different riders' reactions, responds, and riding behaviour change when they ride approaching the speed table. From the study of these collected riding datasets, traffic engineers might get some ideas and pictures on usual riding manner and this may help them design a safer traffic calming devices.

## 2. Methodology

### 2.1. Subjects Selection

A total number of 69 riders with motorcycle riding experience were recruited to participate in this study. All recruited riders were voluntary participants in the project. The criteria of rider selection are as follows. (1) They need to have a valid motorcycle license; (2) they need to ride motorcycle regularly, to be precise, at least once a week; and (3) they should ride at motorway in a regular basis. The mean age of participants is 31.35, mean riding experience is 13.26, and mean kilometers' riding per week is 166.21. Riders were requested to ride with their usual riding manner and behaviour on the instrumented motorcycle. Subjective measures and riders' personal details were taken from a simple questionnaire. Basic information and personal details of riders were taken for record and analysis purposes. The subjective measures and personal information which were included in the survey form were rider's name, gender, occupation, age, license class owned, years of riding experience, and average weekly travel mileage.

### 2.2. Experimental Vehicle

Subjects performed all experimental sessions within an instrumented Honda Wave 110. This motorcycle contained various sensors apparatus such as steering angle sensor, throttle sensor, pedal position sensor which is linked to the brake pedal, steering folk, and throttle roll. The registered speed and distance travelled were recorded using GPS loggers that were fitted inside the motorcycle storage box. A GPS antenna is installed at the highest point of the motorcycle to ensure that the GPS signal can be received clearly throughout the experimental riding process. The GPS signal give all the GPS important information such as the motorcycle GPS coordinate, riding speed, latitude, altitude, curve radius, and other information. All data were sampled at the frequency of 100 Hz, digitized, and stored on a compact flash card which was fitted into the video logger.

**Figure 1:**
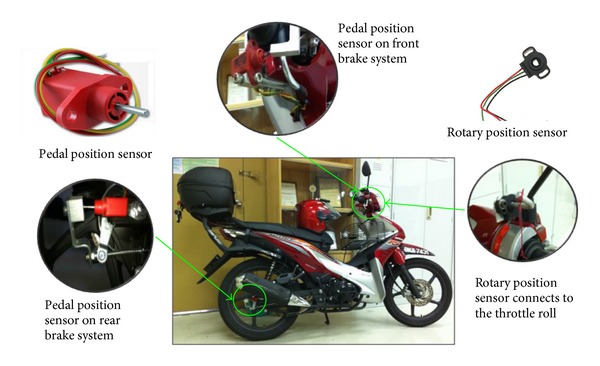
Snapshot of the instrumented motorcycle.

**Figure 2:**
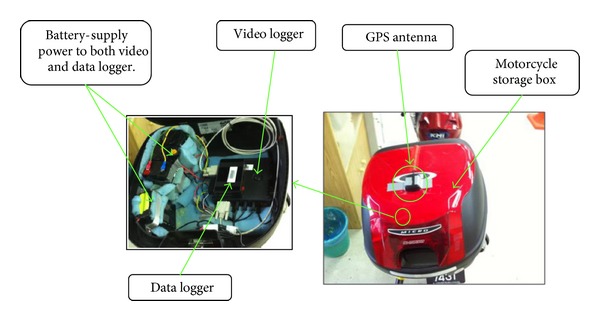
Installation of data logger, video logger, and GPS antenna on the instrumented motorcycle.

On the instrumented motorcycle, a sensor was installed both at the front brake and rear brake pedal to measure the intensity of brake force applied. Once the brake pedal is applied, the braking force measured is converted and presented in percentage reading. Both brake system values are recorded and combined as one overall brake value. In this case, the higher value of the rear brake and front brake will be taken as the overall brake value. In other words, the brake value is taken as the maximum value from either the rear brake or the front brake intensity force. Besides, there were three bullet cameras used to capture real-time traffic image while running the test. One bullet camera is mounted on the riders' helmet to provide their real-time view image. Another bullet camera is mounted in front of the motorcycle to capture the front traffic stream. The third bullet camera is mounted on the back of the motorcycle to capture the real-time back traffic stream. All video streams will be transferred and stored in a video logger. Figures 1 and 2 showed the arrangement of sensors and data loggers on the instrumented motorcycle.

### 2.3. Study Sites Selection

**Figure 3:**
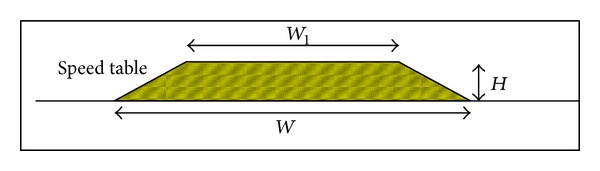
Geometrical cross section of speed table.

In this research, we wanted to focus on the change in riding behaviour of different riders, provided that the other aspects are as similar as possible, for instance, having the same traffic condition, weather condition, and even the same motorcycle. In short, this research focuses on studying the change of riding behaviour from different riders under the same circumstances. Thus, two speed tables in different sites with similar geometrical design and dimension are taken for this study. The chosen sites are located in the main campus of University of Malaya, Kuala Lumpur, and with good pavement surface with traffic moving under free flow conditions. Besides, there should not be any other obstructions or interferences such as a hump or junction at a distance of 100 m before the studied humps. This criterion is very important as we wanted to make sure that riders can ride at a free speed at the initial distance (100 meters) from the hump, which also enables us to obtain a more accurate riding behaviour result, completely free from other outside interference. The reason to select the route within the university campus is that riders can ride without being influenced by other elements such as heavy traffic flow, traffic congestion, and interference of traffic lights. Besides, the safety of the participants can be secure as they ride in a safer road environment within the university campus. The cross sectional sketches and hump dimension design for the selected speed tables were shown in Figure 3 and Table 1.

A total number of 138 data sets were collected for the speed table riding study. Markers were made at the important points such as the initial point (100 m before hump), on the hump point, and 50 m after the hump crossing on the virtual route map for analysis purposes. The traffic data were collected starting at the 100 m point before the hump till the 50 m point after the hump crossing. The reason for taking 100 m before the hump as a starting point is because this is an appropriate distance to cover for riders travelling at a free speed and a good distance to observe when a hump starts to take an impact on reducing riders' speed and influencing riding behaviour in response to the hump.

### 2.4. Experimental Riding Procedures

Each experimental riding session was conducted according to the following protocol: on arrival at the starting point, the subjects were briefed on the route map. Subjects were instructed to ride on a motorway route within the campus of University of Malaya, Malaysia. The selected route covers two sites of speed table. The subjects were asked to ride with their usual and natural riding behaviour. Riders were requested to take one riding section on the selected route which consists of two speed tables with similar geometric design. Thus, a total number of 138 data sets of riding data were collected from two speed tables riding. All the riding sessions were performed in the morning period, when the weather was clear and the roadway was in dry condition. Upon completion of the journey, the subjects were asked to complete a simple questionnaire. Subjective measures such as age, riding experience, and others were taken from the simple questionnaire for record and analysis purposes. The traffic data collected in the experimental run was recorded and stored in a compact flash memory card. The data was then further analysed using system manufacturer analysis software. From the analysis software, data such as travel speed, throttles applied, brakes applied and GPS positions of the motorcycle were extracted.

## 3. Results and Discussions

### 3.1. Speed Analysis

**Figure 4:**
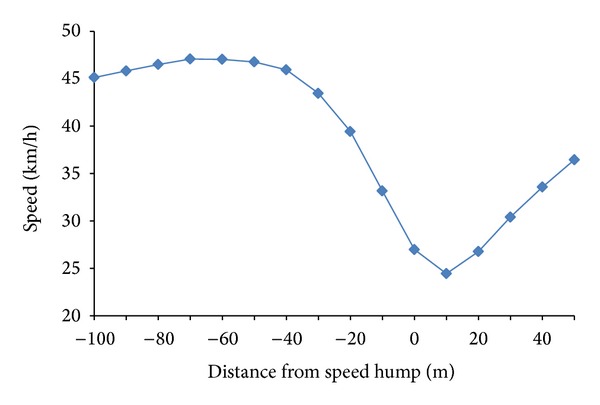
Mean speed profile at speed table riding.

In the analysis section, all the collected riding data, which is 138 sets in total from both STA and STB, were used in conducting the data analysis. The details of the analysis results are shown in Figure 4.

**Figure 5:**
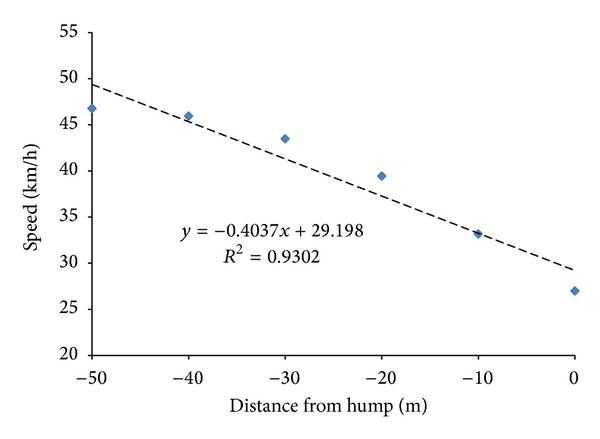
Scatter plots of speed versus distance from speed table.

As observed from Figure 4, the mean riding speed was found increased from the initial point (−100 m) and peaked (47.074 km/h) at distance point −70 m but started to drop at point −60 m from the speed table. From the point −60 m onwards, speed was found negatively correlated with the distance from hump. Interestingly, the minimum riding speeds were found at the point not on the hump but at 10 m after the hump, which was against our presumption that the minimum speed would exist right on the speed table. Besides, it was found that starting from the point −50 m prior to the speed table, the mean speed value can be predicted by a linear regression line as shown in Figure 5. The coefficient of correlation for the linear trend line was found as 0.930, while the linear regression equation is

(1) $$\begin{array}{c} \text{Speed}=-0.4037\;\text{Distance}+29.198. \end{array}$$

From the above observation, we can conclude that the speed table has the impact on the speed reduction starting from the point −60 m distance from the hump. Besides, when the speed variation rate (Table 2) was studied, it was found that the rate value rose gradually from −0.036 km/h (−60 m) to −6.182 km/h (0 m). This indicated that speed drops in much higher rate when they ride approaching to the speed table and reach the minimum speed (24.445 km/h) at the point 10 m after the speed table. It was found that the speed had reduced by 22.321 km/h or 47.73%, from the point −50 m to the point 10 m. After the point 10 m, riders started to accelerate and increased the speed at a rate of more than 2 km/h. Next, when study on the speed class frequency table was carried, it was observed that 50–60 km/h speed class was the second dominant class after 40–50 km/h speed class from the point −100 m till the point −40 m. It showed that most riders were travelling at higher speed at further distances away from the speed table. However, at the point 30 m before the speed table, 30–40 km/h speed class has overtaken and become the second dominant class after 40–50 km/h and eventually become the most dominant class at the final 20 m prior to the speed table. In short, riders still ride at usual speed at the distance 60 m prior to the speed table. However, the speed started to drop from the point 60 m onwards and reached minimum value at 10 m after crossing the speed table. In other words, speed table had effectively reduced the riding speed started from the distance point 60 m before the speed table.

**Figure 6:**
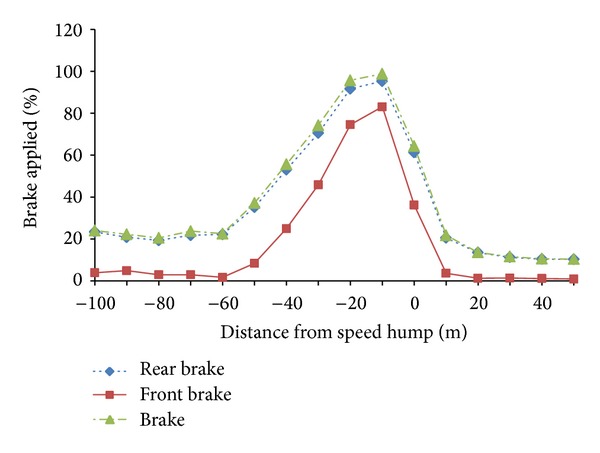
Brake applied profile at speed table riding.

As observed from Figure 6, riders were found tending to apply higher rear brake compared to the front brake. Wilcoxon test was performed to study whether there was significant difference between rear brake and front brake force applied in this section. From the analysis results, *Z* = −21.641, *N* = 1034, and *P* < 0.001, which indicate that the brake force applied for both systems were found to be significantly different. Besides, results also reveal that difference of the brake value (front brake - rear brake) was found as 832 cases at negative rank, 202 cases at positive rank, while 70 cases at equal rank. This again showed that in most distance points, rear brake applied values were found higher compared to the front brake applied values. As what we observed from Figure 4 again, generally, in the first 40 m from the initial point, riders were found to apply very low brake force. Starting from the distance point −60 m, brake force applied rose gradually and peaked (64.33%) at the distance point 10 m before the speed table. The brake force intensity then dropped drastically to below 20% right after they ride past the speed table.

Table 3 illustrates the frequency values on different brake intensity classes in various distances from the speed table. When further analysed from the table, it revealed that most riders applied brake within two classes, which were below 20% brake intensity class and above 80% brake intensity class. Starting from the initial point (−100 m) to the point −50 m, it was found that most riders applied brake intensity at below 20%, where the frequency percentage ranged from 49.28% to 68.12%. From the distance point −40 m onwards, over 80% brake intensity class had become the dominant group as the frequency values were found as 46.38% and peaked at the point −10 m, where 97.10% riders were found to apply brake value within this class. After they ride past the speed table, it is logic that most riders were no longer found to apply brake force and the brake intensity applied had drop back to below 20%. Taking together the observation and analysis obtained from the results above, we can conclude that riders applied higher brake intensity to decrease the riding speed starting from the distance point 50 m before the speed table.

#### 3.1.1. Structural Equation Modeling

**Figure 7:**
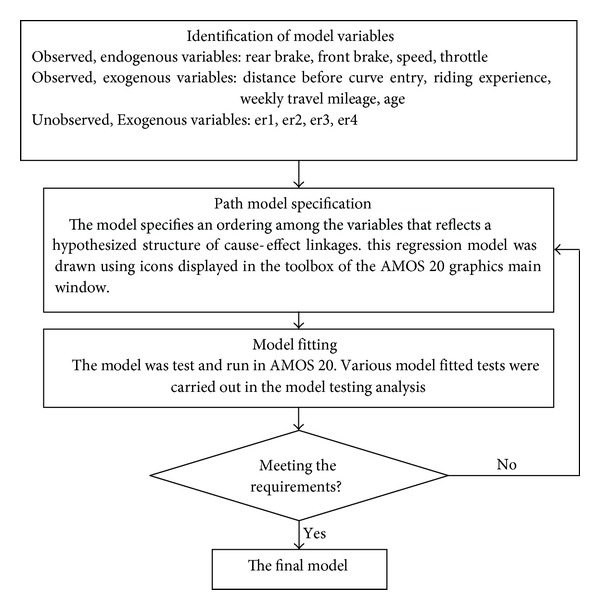
Framework showing the process of the development of SEM for riding behaviour before curve entry.

**Figure 8:**
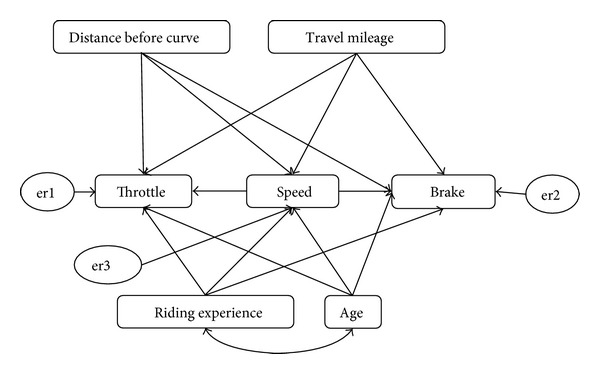
Path model for the prediction of riding behaviour before speed table.

The model was developed on the basis of structural equation modeling (SEM). Structural equation modeling is a multivariate technique that can be described as a combination of both factor analysis and path analysis. SEM is able to estimate the multiple and interrelated dependence relationship simultaneously. Besides, conventional multiple regression technique assumes that the variables in the analysis are error-free, while SEM improves statistical estimation by accounting for measurement error in the estimation process. In short, SEM analysis removed the potential biasing effects of the measurement error on the results, thus improving the statistical estimation process. Furthermore, from the SEM model, we are able to identify the impact factors for each predictor towards the dependent variables and this is important to study the effect of different riders' attributes on the riding behavior change. The process of development SEM path model for the study is shown as in Figure 7, while Figure 8 illustrates the final path model for the entire riding behaviour study on curve entry.

Table 4 above showed the list of variables that are contained in the model. The input covariance matrix generated from the model contains 35 sample moments. For the hypothesized model (Figure 5), there are 29 parameters to be estimated. The model, therefore, has positive degrees of freedom (35 − 29 = 6), and the chi-squared goodness-of-fit was computed. The result indicates that the model did not fit the data well by chi-square test, *χ*2(*N* = 414, df = 6) = 190.211, *P* < 0.05. Although the hypothesized model did not fit the observed variance-covariance matrix well by the chi-square test, the baseline comparisons fit indices of Normal Fit Index (NFI), Relative Fit Index (RFI), Incremental Fit Index (IFI), Tucker-Lewis Index (TLI), and Comparative Fit Index (CFI) all range from 0.652 to 0.903 (Table 5). These indices compare the fit of the hypothesized model to the null or independence model. They range from 0 which means a fit that is no better than the null model to 1 which is a perfect fit. Given the range of the computed baseline comparisons fit indices, the model sufficiently fit the data.

The covariance between “age” and “riding experience” is found highly significant by CR test (*P* < 0.001). The standardized correlation coefficient is found as 0.971, as shown in Table 6. Squared multiple correlation is an index of the proportion of the variance of the endogenous variable that is accounted for by the exogenous of predictor variables. It can be assumed that the higher the value of squared multiple correlation, the greater the explanatory power of the regression model and therefore the better the prediction of the dependent variable. Squared multiple correlations table showed that the percentage of variance explain ranged from 0.126 or 12.6% (throttle) to 0.609 or 60.9% (speed). Since the original path model consists of three regression models, it is easier to study if we discuss and explain the model separately.

Standardized coefficient estimate (*β*) is independent of the units in which all variables are measured. These standardized coefficients allow the researcher to compare directly the relative relationship between each independent variable and the dependent variable. In the speed regression model, it was found that the standardized regression weights are all significant by the critical ratio test (>±1.96; *P* < 0.05) (see Table 6). It can be seen that ratings on three variables of “distance,” “age,” and “travel mileage” are both significantly and positively correlated to “speed” (*β* = 0.756; *β* = 0.435; *β* = 0.075, resp.), while “riding experience” is found negatively correlated to the “speed.” Thus, it can be concluded that the speed had reduced when the riders approach the curve entry. While in the brake regression model, only the “distance” and “speed” were significant predictors by the critical ratio test, when the *P* is below 0.05. Since the probability value for other predictors was over 0.05, there was no proof of significant influence of these predictors towards brake applied. Thus, these predictors were excluded from the regression model. The “distance” predictor was found as negatively correlated with the brake force applied (*β* = −0.604), while the “speed” was found as positively correlated with brake force (*β* = 0.286). This finding indicated that the higher the speed is and the closer the riders are to the speed table, the higher brake force intensity was applied. In the throttle regression model, the standardized regression weights are all significant by the critical ratio test (>±1.96, *P* < 0.05) except for “riding experience” and “age.” Thus, the “riding experience” and the “age” were not included in the regression model. Besides, it was found that the “speed” and the “travel mileage” were negatively correlated with the throttle applied, while the “distance” was positively correlated with the throttle. This indicated that lower throttle force was applied when in higher travel speed and at the distance closer to the speed table. The standardized regression equation for these models was shown as follows:

(2) Speed=0.756  Distance+0.435  Age−0.571  Riding  Exp.+0.075  Travel  Mileage,Brake=−0.604  Distance+0.286  Speed,Throttle=0.462  Distance−0.278  Speed−0.120  Travel  Mileage.

## 4. Conclusion

The present study was designed to determine the effects of a speed table on a motorcyclist's riding speed as well as throttle and braking behaviour. The evidence from this study suggests that there is a significant correlation between various aspects of riding behaviour such as speed, throttle, and brakes applied, with riders' attributes and distance from the speed table. In general, riders rode with their usual riding speed at the initial point but started to reduce their speed at the distance 60 m before the speed table and reach minimum speed at the point 10 m after the speed table. While in the brake analysis study, in most cases, riders were found tending to apply higher rear brake compare to front brake. Furthermore, riders were found applying higher brake intensity at the distance 50 m prior to the speed table and released the throttle force to achieve a comfortable speed for the speed table crossing. Structural equation modeling (SEM) analysis is used to develop a model which suits the collected data in our case study. The SEM model on speed had revealed that there are four significant factors, which are distance, age, riding experience, and travel mileage. While for the SEM regression model on brake, brake was found to have direct negative correlation with the distance and direct positive correlation with the approaching speed. In the throttle model, “distance,” “approach speed,” and “travel mileage” were found as the significant factors with various impact rates in the throttle force applied.

The instrumented motorcycle is a very powerful tool as it provided a robust, accurate, and reliable way of collecting such important motorcycle riding data in this study of motorcyclist riding behaviour. Each year numbers of motorcyclists died in road crashed in Malaysia. One of the reasons that caused the high rates of accident involving motorcycle is that people may not fully understand how and why motorcycle accidents actually happen. By conducting such motorcycle accident and riders' behaviour studies, traffic engineers, law enforcers, and policy makers may take effective actions to reduce the number of accidents and therefore save valuable human life.

## Figures and Tables

**Table 1:** Geometrical dimension design for speed table.

Hump mark	Width, *W* (cm)	Width, *W*_1_ (cm)	Height, *H* (cm)
ST A	640	400	12.5
ST B	550	300	12.5

**Table 2:** Speed class frequency, mean speed, and differences in various distances from speed table.

Distance (m)	Frequency percentage of riding speed class					Mean speed (km/h)	Difference (km/h)
	≤30	30–40	40–50	50–60	>60		
−100	0.00%	26.09%	47.83%	26.09%	0.00%	45.125	—
−90	0.00%	20.29%	50.72%	27.54%	1.45%	45.820	0.695
−80	1.45%	17.39%	49.28%	31.88%	0.00%	46.497	0.677
−70	1.45%	15.94%	49.28%	31.88%	1.45%	47.074	0.577
−60	1.45%	15.94%	49.28%	31.88%	1.45%	47.038	−0.036
−50	1.45%	14.49%	53.62%	30.43%	0.00%	46.766	−0.272
−40	0.00%	17.39%	53.62%	27.54%	1.45%	45.938	−0.828
−30	1.45%	21.74%	68.12%	8.70%	0.00%	43.460	−2.479
−20	4.35%	50.72%	43.48%	1.45%	0.00%	39.430	−4.029
−10	15.94%	78.26%	5.80%	0.00%	0.00%	33.162	−6.268
0	75.36%	21.74%	2.90%	0.00%	0.00%	26.980	−6.182
10	82.61%	17.39%	0.00%	0.00%	0.00%	24.445	−2.535
20	84.06%	14.49%	1.45%	0.00%	0.00%	26.771	2.325
30	50.72%	46.38%	2.90%	0.00%	0.00%	30.412	3.641
40	13.04%	84.06%	2.90%	0.00%	0.00%	33.572	3.161
50	2.90%	78.26%	18.84%	0.00%	0.00%	36.455	2.882

**Table 3:** Frequency percentage on brake applied in various distances from speed table.

Distance (m)	Frequency percentage on riding brake class				
	≤20	20–40	40–60	60–80	>80
−100	66.67%	2.90%	13.04%	7.25%	10.14%
−90	66.67%	4.35%	14.49%	5.80%	8.70%
−80	68.12%	10.14%	8.70%	5.80%	7.25%
−70	60.87%	14.49%	8.70%	10.14%	5.80%
−60	63.77%	8.70%	10.14%	11.59%	5.80%
−50	49.28%	7.25%	8.70%	14.49%	20.29%
−40	36.23%	4.35%	2.90%	10.14%	46.38%
−30	10.14%	14.49%	7.25%	5.80%	62.32%
−20	1.45%	1.45%	0.00%	2.90%	94.20%
−10	0.00%	0.00%	0.00%	2.90%	97.10%
0	7.25%	8.70%	23.19%	27.54%	33.33%
10	66.67%	13.04%	10.14%	5.80%	4.35%
20	73.91%	8.70%	13.04%	2.90%	1.45%
30	75.36%	10.14%	13.04%	0.00%	1.45%
40	78.26%	8.70%	11.59%	0.00%	1.45%
50	79.71%	7.25%	11.59%	0.00%	1.45%

**Table 4:** List of model variables.

Observed, endogenous variables	Observed, exogenous variables	Unobserved, exogenous variables
Speed	Weekly Travel Mileage	er1
Throttle	Distance before speed table	er2
Brake	Riding experience	er3
	Age	

**Table 5:** Baseline comparisons fit indices.

NFI Delta 1	RFI rho 1	IFI Delta 2	TLI rho 2	CFI
0.901	0.652	0.903	0.660	0.903

**Table:** (a) Regression weights and standardized regression weights

			Unstandardized coefficient est.	Standardized coefficient est.	S.E.	C.R.	*P*
Speed	←	Travel mileage	0.003	0.075	0.001	2.426	0.015
Speed	←	Distance	0.404	0.756	0.016	24.568	- - - - -
Speed	←	Riding exp.	−0.489	−0.571	0.111	−4.427	- - - - -
Speed	←	Age	0.379	0.435	0.112	3.371	- - - - -
Throttle	←	Distance	0.216	0.462	0.034	6.395	- - - - -
Brake	←	Distance	−1.276	−0.604	0.143	−8.923	- - - - -
Throttle	←	Travel mileage	−0.005	−0.120	0.002	−2.582	0.010
Brake	←	Travel mileage	0.005	0.026	0.008	0.608	0.543
Throttle	←	Riding exp.	−0.221	−0.295	0.148	−1.492	0.136
Brake	←	Riding exp.	−1.217	−0.359	0.627	−1.940	0.052
Throttle	←	Speed	−0.243	−0.278	0.064	−3.781	- - - - -
Brake	←	Speed	1.130	0.286	0.273	4.140	- - - - -
Throttle	←	Age	0.275	0.361	0.149	1.848	0.065
Brake	←	Age	0.710	0.206	0.632	1.123	0.261

**Table:** (b) Variance

			Covariances	Correlation coefficient	S.E.	C.R.	*P*
Riding exp.	↔	Age	108.226	0.971	7.644	14.158	∗∗∗

**Table:** (c) Squared multiple correlations

			Estimate
Speed			0.609
Brake			0.232
Throttle			0.126
